# A Comparison of the Beneficial Effects of Live and Heat-Inactivated Baker’s Yeast on Nile Tilapia: Suggestions on the Role and Function of the Secretory Metabolites Released from the Yeast

**DOI:** 10.1371/journal.pone.0145448

**Published:** 2015-12-22

**Authors:** Chao Ran, Lu Huang, Zhi Liu, Li Xu, Yalin Yang, Philippe Tacon, Eric Auclair, Zhigang Zhou

**Affiliations:** 1 Key Laboratory for Feed Biotechnology of the Ministry of Agriculture, Feed Research Institute, Chinese Academy of Agricultural Sciences, Beijing, People’s Republic of China; 2 Société Industrielle Lesaffre, Phileo Lesaffre Animal Care, Marcq-en-Baroeul, France; Catalan Institute for Water Research (ICRA), SPAIN

## Abstract

Yeast is frequently used as a probiotic in aquaculture with the potential to substitute for antibiotics. In this study, the involvement and extent to which the viability of yeast cells and thus the secretory metabolites released from the yeast contribute to effects of baker’s yeast was investigated in Nile tilapia. No yeast, live yeast or heat-inactivated baker’s yeast were added to basal diets high in fishmeal and low in soybean (diet A) or low in fishmeal and high in soybean (diet B), which were fed to fish for 8 weeks. Growth, feed utilization, gut microvilli morphology, and expressions of *hsp70* and inflammation-related cytokines in the intestine and head kidney were assessed. Intestinal microbiota was investigated using 16S rRNA gene pyrosequencing. Gut alkaline phosphatase (AKP) activity was measured after challenging the fish with *Aeromonas hydrophila*. Results showed that live yeast significantly improved FBW and WG (*P* < 0.05), and tended to improve FCR (*P* = 0.06) of fish compared to the control (no yeast). No significant differences were observed between inactivated yeast and control. Live yeast improved gut microvilli length (*P* < 0.001) and density (*P* < 0.05) while inactivated yeast did not. The *hsp70* expression level in both the intestine and head kidney of fish was significantly reduced by live yeast (*P* < 0.05) but not inactivated yeast. Live yeast but not inactivated yeast reduced intestinal expression of *tnfα* (*P* < 0.05), *tgfβ* (*P* < 0.05 under diet A) and *il1β* (*P* = 0.08). Intestinal *Lactococcus* spp. numbers were enriched by both live and inactivated yeast. Lastly, both live and inactivated yeast reduced the gut AKP activity compared to the control (*P* < 0.001), indicating protection of the host against infection by *A*. *hydrophila*. In conclusion, secretory metabolites did not play major roles in the growth promotion and disease protection effects of yeast. Nevertheless, secretory metabolites were the major contributing factor towards improved gut microvilli morphology, relieved stress status, and reduced intestinal inflammation of Nile tilapia fed diets supplemented with baker’s yeast.

## Introduction

Aquaculture remains the fastest growing agri-business sector with total global production (inland and marine, without aquatic plants) increasing from 47.3 million tons in 2006 to 70.2 million tons in 2013 [1]. Antibiotics have been widely used for growth promotion and disease control in aquaculture practices. However, antibiotics usage is associated with problems such as bioaccumulation and development of antibiotic-resistant pathogens [2]. As a result, the European Union has banned the use of antibiotics in animal feed as growth promoters in 2006, and the FDA has introduced measures to ensure the judicious use of antibiotics in food-producing animals in 2012. Thus there has been an increasing interest in developing novel agents or functional dietary supplements to improve growth and health in fish.

Probiotics have received a great deal of attention as novel dietary supplements to replace antibiotics in aquaculture [3, 4]. In particular, yeast, *Saccharomyces cerevisiae*, has been used as a probiotic in various aquatic species and the beneficial effects reported include growth promotion, enhancement of innate immune response, as well as protection against pathogen infection [5–10]. The beneficial effects of yeast have often been attributed to the structural components in the yeast cell wall, such as β-glucans and mannan oligosaccharides (MOS), due to their immunostimulating or gut-health promoting effects [6, 11, 12]. Yeast also produces various metabolites such as enzymes, oligosaccharides, amino acids, peptides, organic acids, vitamins, and other soluble factors. Various metabolites of yeast have been proposed to contribute to improved growth performance of the broiler chicks [13]. Effects of amino acids, peptides, and vitamins on immune function have been reported in mammals [14–16]. In addition, the soluble fraction of yeast cultures exhibited anti-inflammatory effects in swine intestinal epithelial cell lines and immune-modulating effects in NK cells and B-lymphocytes *in vitro* [17, 18]. The secretion of metabolites by yeast was suggested to rely on yeast viability [19], and the influence of yeast viability was used to deduce the contribution of secreted factors linked to the anti-inflammatory effect of yeast [17]. Dietary inactive yeast has been investigated on different fish species, with beneficial effects reported in growth performance, disease resistance, and gut microbiota [20–22]. However, no direct comparison of the probiotic effects of live and inactivated yeast in fish has been conducted.

In this study, heat-inactivated baker’s yeast was prepared. The effect of live and heat-inactivated yeast on growth performance, gut morphology, immune response, intestinal microbiota, as well as disease resistance of the economically important Nile tilapia was compared to evaluate the contribution of secretory metabolites to the mode of action of the yeast. Two basal diets with different levels of fishmeal and soybean meal similar to those used in the industry were used to investigate the potential interactions of the yeast (live *vs* inactivated) with diet.

## Experimental Methods

### Experimental diets

Two basal diets were formulated, representative of current practices aimed at reducing fishmeal incorporation rates: one diet with relatively higher fishmeal and lower soybean content (diet A) and the other with relatively lower fishmeal and higher soybean content (diet B). Each basal diet was then kept as control (CK) or supplemented with dietary live yeast (LY) or its heat-inactivated counterpart (HIY). The live yeast, *Saccharomyces cerevisiae*, was obtained as commercial preparation, Actisaf^®^ (Lesaffre, France). The experimental diet was incorporated with 1 g/kg of the Actisaf^®^ yeast for a final concentration of 10^7^ CFU/g of feeds. Heat-inactivated yeast was prepared by incubating Actisaf^®^ yeast at 80°C for 30 min. After the heat treatment, the yeast preparation was washed with PBS for three times to get rid of potential secretory metabolites present in the heat-inactivated yeast preparation. The effectiveness of the heat treatment was confirmed by attempting to culture on YPD agar, with no yeast colonies growing on the plates. Heat-inactivated yeast was also supplemented as 1 g/kg of the feed. Consequently, a total of six experimental diets were formulated, i.e., CKA, LYA, HIYA, CKB, LYB, and HIYB, representing control, live yeast supplemented diet, and heat-inactivated yeast supplemented diet for diet A and B, respectively. The formulation and chemical composition of the experimental diets (%) are presented in Table 1. The dietary ingredients were blended with 100 ml of water per 1 kg of diet to form a paste which was then passed through a meat grinder equipped with a 3-mm die to obtain uniform pellets. The pelleted diets were air-dried till the moisture content was reduced to less than 10%. The levels of *S*.*cerevisiae* in diets LYA and LYB were verified by pellet homogenization, serial dilution followed by culturing of the dilutions on YPD plates. All the diets were stored in plastic bags at 4°C until use.

**Table 1:** Formulations and chemical compositions of the experimental diets.

Ingredients (%)	Basal diet of soybean meal type					
	CKA	LYA	HIYA	CKB	LYB	HIYB
Soybean meal	14.0	14.0	14.0	28.0	28.0	28.0
Cottonseed meal	10.0	10.0	10.0	10.0	10.0	10.0
Zeolite powder	2.95	2.85	2.85	1.48	1.38	1.38
Adhesives	0.20	0.20	0.20	0.20	0.20	0.20
VC phosphate ester	0.05	0.05	0.05	0.05	0.05	0.05
Mineral Premix	0.20	0.20	0.20	0.20	0.20	0.20
Vitamin Premix	0.20	0.20	0.20	0.20	0.20	0.20
Lysine Sulphate	0.22	0.22	0.22	0.19	0.19	0.19
Calcium methionine hydroxy	0.16	0.16	0.16	0.28	0.28	0.28
Calcium dihydrogen phosphate	2.00	2.00	2.00	2.00	2.00	2.00
Bean oil	4.02	4.02	4.02	4.40	4.40	4.40
Rapeseed meal	28.0	28.0	28.0	20.0	20.0	20.0
Flour	24.0	24.0	24.0	24.0	24.0	24.0
Domestic fish meal (mixed)	10.0	10.0	10.0	5.00	5.00	5.00
Corn gluten meal	4.00	4.00	4.00	4.00	4.00	4.00
Live yeast	0	0.10	0	0	0.10	0
Heat-inactivated yeast	0	0	0.10	0	0	0.10
**Estimated chemical compositions (%)**						
Crude protein	31.9	31.9	31.9	32.4	32.4	32.4
Crude lipid	6.73	6.73	6.73	6.83	6.83	6.83
Crude ash	6.38	6.38	6.38	5.50	5.50	5.50
Crude fiber	5.95	5.95	5.95	6.84	6.84	6.84

### Fish and rearing conditions

All experimental and animal care procedures were approved by the Feed Research Institute of Chinese Academy of Agricultural Sciences Animal Care Committee, under the auspices of the China Council for Animal Care (Assurance # 2012 ZZGCC01). MS-222 was used as the anaesthetic. Nile tilapia, *Oreochromis niloticus* (L.) fingerlings were obtained from the local aquaculture farm in Beijing, China. After disinfection with 2‰ salt solution, the fish were acclimatized in aquaria (50 ×30 × 38 cm) in a recirculation system (at 0.5 L/min) for at least 2 weeks before the feeding trial. Fish of a similar size (0.66 g) were randomly chosen and distributed into 24 tanks at a density of 12 fish per tank. Each diet was randomly assigned to four tanks and hand-fed to apparent satiation three times daily (8:00, 11:30 and 17:30). During the feeding trial, water temperature and pH were maintained in the range of 25.4 ± 1.68°C and 7.7 ± 0.1, respectively; dissolved oxygen was higher than 5.0 mg O/L; NH_4_^+^-N and NO_2_^−^-N were lower than 0.50 mg N/L and 0.05 mg N/L, respectively. The photoperiod was 12 h light and 12 h dark, with the light period from 08:00 to 20:00.

### Growth performance and sampling

After 8 weeks feeding, all the fish were batch weighed and counted. Two fish were randomly chosen from each tank and anaesthetised with MS-222 (50.0 mg/l). The intestine was sampled. A piece of the midgut (~1 cm) was cut and gently agitated three times in PBS (pH 7.2) for 1 min to remove the digesta for intestinal mucosal morphology investigation; the rest of midgut was washed the same way and used for RT-PCR analysis. The hindgut was used for analysis of autochthonous and allochthonous microbiota. The head-kidney was also sampled for RT-PCR analysis. Weight gain (WG) and Feed conversion ratio (FCR) were calculated as follows:

$$\text{Weight gain}\;\left(\text{WG}\;\%\right)=\frac{\text{final weight}(\text{g})\text{-initial weight}(\text{g})}{\text{initial weight}(\text{g})}\times 100$$

$$\text{Feed conversion ratio}\;\left(\text{FCR}\right)=\frac{\text{total feed consumption}(\text{total feed casting -total food residue})(\text{g})}{\text{total final weight}(\text{g})\text{-total initial weight}(\text{g})+\text{total mora;ity weight}(\text{g})}$$

### Intestinal microvilli morphology

Intestine samples were processed for transmission electron microscope (TEM) scanning electron microscopy (SEM) analysis according to the methods described by Liu *et al*. [23]. In brief, the midgut intestine was fixed with 2.5% glutaraldehyde. The segments were washed twice in phosphate buffer (pH 7.2) and then post-fixed in OsO4 (1% in pH 7.2 phosphate buffer, 2 h). After serial dehydration in alcohol from 30% to 100%, the segments were critical-point dried (BAL-TEC, CPD030, Balzers, Liechtenstein), mounted on aluminum stubs, sputter-coated with gold by using a high-resolution fine coater (BAL-TEC, SCD005, Balzers, Liechtenstein), and then examined under a Jeol JSM-6301 (JEOL, Tokyo, Japan) scanning microscope. For each group, samples of eight replicate fish were processed and analyzed, with each fish giving one TEM/SEM image. TEM images (magnification **×** 30,000) were analyzed to measure the microvilli length. SEM images (magnification **×** 20,000) were used to measure the microvilli density. For the density calculation, ten 1μm ×1μm zoomed squares were randomly taken from each SEM image by PhotoShop CS6 (Adobe, USA), and the microvilli number in each zoomed square was counted by ImageJ (National Institutes of Health, USA). The density data for each fish was the average of the counted microvilli numbers for the ten zoomed squares.

### Expression of *hsp70* and inflammation-related cytokine genes

Total RNA from the intestine and kidney was extracted using a TRIzol Reagent RNA kit (Promega, Germany), and the integrity of total RNA was verified by visualization on a 1.2% agarose gel. RNA was dissolved in 50 μl RNase-free water and stored at − 70°C until use. cDNA was synthesized for quantitative reverse transcription PCR (RT-*q*PCR) using the ReverTra Ace-a-RT-PCR kit (TRAN, Beijing, China) according to the manufacturer's instructions. The *q*PCR primers were designed using Primer 5.0 software based on the available cytokine sequences in GenBank (S1 Table). Additional dissociation curve analysis was performed and showed a single melting curve in all cases. *q*PCR was performed with the SYBR Green SuperReal PreMix Plus (TIANGEN, Beijing, China) in ABI Step OnePlus fluorescence ration PCR instrument (ABI, USA). The total volume of the PCR reactions was 20 μl and consisted of: 10 μl SYBR Green SuperReal PreMix Plus (2 ×), 1μl primer of each, 2μl cDNA, and 6μl RNase-free H_2_O. The cycling conditions were as follows: 95°C for 10 min and then 40 cycles of 95°C for 20 s, 58°C for 20 s, and 72°C for 20 s. All *q*PCRs were performed at least two times. Data analysis was conducted using the 2^-ΔΔCT^method [24], and the β-actin gene (S1 Table) was chosen as the internal standard.

### Gut alkaline phosphatase activity after challenge

After the feeding trial, four replicate tanks of each dietary treatment were infused with a pathogenic strain *A*. *hydrophila* NJ-1 (a gift from Dr. Yongjie Liu from Nanjing Agricultural University) at a concentration of 10^8^ CFU/ml. After 24h, 2 fish each tank were randomly chosen and anaesthetised with MS-222 (50.0 mg/l). The whole intestine was sampled and gently agitated three times in PBS (pH 7.2) for 1 min to remove the digesta. The alkaline phosphatase activity was determined according to Qin *et al*. [25]. In brief, the whole intestine was homogenized in PBS and centrifuged at 8000 rpm, 4°C to obtain the supernatant, which was incubated in a p-nitrophenylphosphate liquid substrate system (Sigma) for 30 min following the manufacturer’s specifications. The absorbance was measured at 405 nm. The concentration of total protein was measured using a BCA Protein Assay Kit (Novagen, Darmstadt, Germany) following the manufacturer’s specifications.

### 16S rRNA gene pyrosequencing and analysis

The gut wall and content of the hindgut were separated for analysis of autochthonous and allochthonous microbiota, respectively. The DNA was extracted from samples according to Liu et al. [26]. Bacterial 16S rRNA genes were amplified by PCR from DNA samples using barcoded primers targeting the V3 -V4 region of 16S *r*RNA gene of bacteria (S1 Table). All forward core primers (‘core’ refers to the original unmodified 16S rRNA amplification primer) were modified by the addition of a PGM sequencing adaptor, a ‘GT’ spacer and unique error correcting Golay barcode [27], to allow multiplex analyses when necessary. Amplification conditions employed were according to previously published protocols [28]. We amplified a standard 450 bp V3-V4 region using the modified primers 349F and 806R. All PCR products were checked for size and specificity by electrophoresis on 1.2% w/v agarose, gel purified and adjusted to 10ng/μl using molecular grade water and pooled equally for subsequent sequencing. V3-V4 amplicons were sequenced using pair-end method by Illumina Hiseq2500. The sequence data are available from the European Nucleotide Archive (ERA) (accession number PRJEB9953).

The raw data was processed and trimmed by FastQC and NGS toolkits. For V3-V4 pair-end reads, only sequences that overlap longer than 10bp and without any mismatch were assembled according to their overlap sequence. Reads with an average base score higher than 30 and base number lower than 80% of this sequence reads were removed by QIIME1.8. Chimera produced in PCR process was removed by USEARCH v6.1. All the reads from fish fed with the same diet (n = 8) were pooled, making 6 final groups, i.e., CKA, LYA, HIYA, CKB, LYB, and HIYB. Sequences were analyzed in UCLUST v1.2.22 to determine Operational Taxonomic Units (OTU), which was defined as comprising sequences with less than a 3% difference by the furthest-neighbor method. Taxon annotation was conducted using one representative sequence from an OTU by RDP classifier referring to GreenGenes database. The relative abundance of OTUs was calculated, and microbial composition in different taxonomic levels was analyzed for each group. Heatmap was constructed for each sample. Species alpha diversity index including Shannon, PD (phylogenetic diversity), good’s coverage was calculated based on normalized data.

### Statistical analysis

Data were subjected to two way ANOVA to test the effects of yeast supplementation, basal diets as well as their interaction. The pyrosequencing data was analyzed with two way ANOVA without replication, and only the main effects of yeast supplementation and basal diets were tested. When the main effect of yeast supplementation was significant or a trend was observed, post hoc testing was performed using Tukey’s test. The effects of yeast supplementation under each basal diet were separately analyzed with one way ANOVA when the interaction was significant. Variance homogeneity of the data was examined with Levene’s test. Differences were considered significant when *P* < 0.05. Instances in which 0.05 < P < 0.1 were discussed as trends. All the statistical analysis was conducted in SPSS 17.0.

## Results

### Growth and feed utilization

The initial body weights in all treatments were very similar. Two way ANOVA showed that both yeast supplementation and basal diet had significant effect on the final body weight, weight gain and feeding conversion ratio (*P* < 0.05) (Table 2). Live yeast significantly improved the FBW and WG of fish compared to the control (*P* < 0.05). Also, a trend for lower FCR was observed in live yeast group relative to the control (*P* = 0.06). The inactive yeast had no significant influence on FBW, WG and FCR of fish, although with numerical improvement compared to the control. No significant difference was observed between the live and inactivated yeast groups. Fish fed diet B showed significantly improved FBW, WG and FCR, compared with the diet A fed fish (*P* < 0.001).There were no significant interactions between yeast and basal diet (*P* > 0.50).

**Table 2:** Effects of yeast supplementation and basal diets on the growth performance and feed utilization of Nile tilapia.

	Yeast			Basal diet			*P* value		
	CK	LY	HIY	A	B	SEM	Yeast	Basal diet	Interaction
FBW/g	12.1^a^	12.6^b^	12.5^ab^	12.0	12.8	0.3	0.04	<0.001	0.97
WG/%	1876^a^	1958^b^	1942^ab^	1866	1984	47	0.04	<0.001	0.98
FCR	1.52	1.46	1.47	1.53	1.44	0.04	0.049	<0.001	0.97

Values represent means of data obtained from 8 and 12 tanks for ‘Yeast’ and ‘Basal diet’ unit cells, respectively. Means sharing a common superscript letter (a, b, ab) were not significantly different (*P* > 0.05). CK, control check; LY, live baker’s yeast; HIY, heat-inactivated yeast; FBW, final body weight; WG, weight gain; FCR, feed conversion ratio.

### Intestinal microvilli morphology

**Fig 1:**
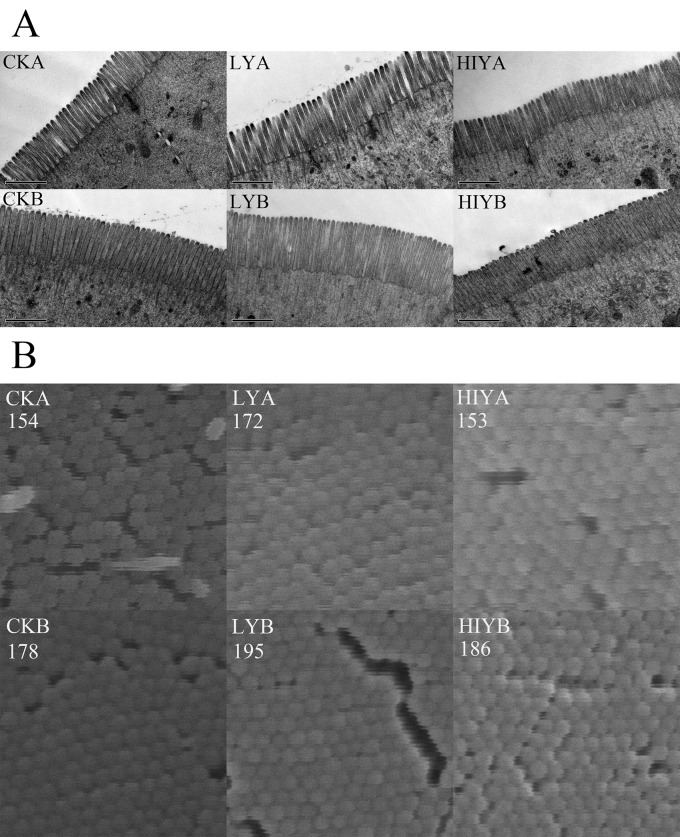
Electron microscope images of the gut microvilli. **(A) The TEM images for microvilli length; (B) The 1μm × 1μm zoomed squares from the original SEM images reflecting the calculation process of the microvilli density.** The counted microvilli number in the zoomed square was put on top left. Each zoomed image was selected such that the average density of the corresponding group can be represented, which involved 8 replicate fish and counting of 10 randomly selected 1μm × 1μm squares for the image of each fish. The original SEM images are in S1 Fig.

Both yeast and basal diet significantly influenced gut microvilli morphology of tilapia (*P* < 0.05) (Table 3, Fig 1, S1 Fig). Live yeast significantly improved microvilli length (*P* < 0.001) and density (*P* < 0.05) compared with control, while inactive yeast failed to exert any beneficial effect on the microvilli morphology. Microvilli length was significantly increased in the live yeast group compared with the inactivated yeast group (*P* < 0.001). Similar with the growth performance results, interactions between yeast supplementation and basal diets were not significant for the microvilli morphology (*P* > 0.10).

**Table 3:** Effects of yeast supplementation and basal diets on intestinal microvilli length and density of Nile tilapia.

	Yeast			Basal diet			*P* value		
	CK	LY	HIY	A	B	SEM	Yeast	Basal diet	Interaction
Length (μm)	0.99^a^	1.10^b^	0.96^a^	0.97	1.07	0.03	<0.001	<0.001	0.17
Density (count/μm^2^)	164.3^a^	181.6^b^	167.8^ab^	156.4	186.0	10.2	0.04	<0.001	0.71

Values represent means of 16 or 24 fish for ‘Yeast’ and ‘Basal diet’ unit cells, respectively. Means sharing a common superscript letter (a, b, ab) were not significantly different (*P* > 0.05). CK, control check; LY, live baker’s yeast; HIY, heat-inactivated yeast.

### Expression of *hsp70* in intestine and head kidney

Live yeast significantly reduced intestinal *hsp70* expression compared to the control (*P* < 0.05). However, the inactive yeast had no significant effect on *hsp70* expression (*P* > 0.05), although the value was numerically lower (Table 4). In head kidney, *hsp70* expression was significantly decreased in the live yeast group compared to the control (*P* < 0.05), while no significant difference was observed in the inactive yeast group (Table 4). In both the gut and head kidney there was no significant interaction between yeast supplementation and basal diet (*P* > 0.20).

**Table 4:** Effects of yeast supplementation and basal dies on the relative expression of *hsp70* and inflammation-related cytokines in the intestine and head kidney of Nile tilapia.

		Yeast			Basal diet			*P* value		
		CK	LY	HIY	A	B	SEM	Yeast	Basal diet	Interaction
	*hsp70*	1.02^a^	0.66^b^	0.80^ab^	0.94	0.72	0.17	0.02	0.04	0.20
Gut	*tnfα*	1.10^a^	0.73^b^	1.12^a^	0.90	1.08	0.20	0.02	0.14	0.42
	*il1β*	1.03	0.72	1.00	1.02	0.81	0.21	0.05	0.05	0.39
	*tgfβ*	1.04	0.82	0.89	0.93	0.90	0.17	0.15	0.86	0.02
	*hsp70*	1.02^a^	0.46^b^	0.77^ab^	0.79	0.70	0.20	0.001	0.43	0.44
HK	*tnfα*	1.03	0.79	0.85	0.89	0.88	0.10	0.10	0.84	0.40
	*il1β*	1.06	0.72	1.04	0.77	1.09	0.26	0.12	0.04	0.17
	*tgfβ*	1.01^a^	0.69^b^	0.73^b^	0.77	0.86	0.13	0.002	0.18	0.47

Values represent means of 16 or 24 fish for ‘Yeast’ and ‘Basal diet’ unit cells, respectively. The units of the values are arbitrary units (a.u.). Means sharing a common superscript letter (a, b, ab) were not significantly different (*P* > 0.05). CK, control check; LY, live baker’s yeast; HIY, heat-inactivated yeast; HK, head kidney.

### Expression of inflammation-related cytokine genes in intestine and head kidney

Live yeast significantly reduced intestinal *tnfα* expression compared to the control and inactivated yeast group (*P* < 0.05). A trend towards decreased intestinal expression of *il1β* (*P* = 0.08) was observed in the live yeast group compared to the control (Table 4). A significant interaction between yeast supplementation and basal diet was observed for the intestinal expression of *tgfβ* (*P* < 0.05). The expression was significantly lower in the live yeast group compared with control and inactivated yeast group when diet A was fed (*P* < 0.05), while no significant effect was observed when diet B was fed (S2 Fig). Inactivated yeast had no significant effects on the cytokine genes expression.

In head kidney, yeast supplementation had no effect on the expression of *tnfα* nor *il1β* (Table 4). The expression of *tgfβ* was significantly down-regulated by both live and inactivated yeast compared to the control (*P* < 0.05). No significant interaction between yeast and basal diet was observed (*P* > 0.10).

### Intestinal autochthonous and allochthonous microbiota

**Fig 2:**
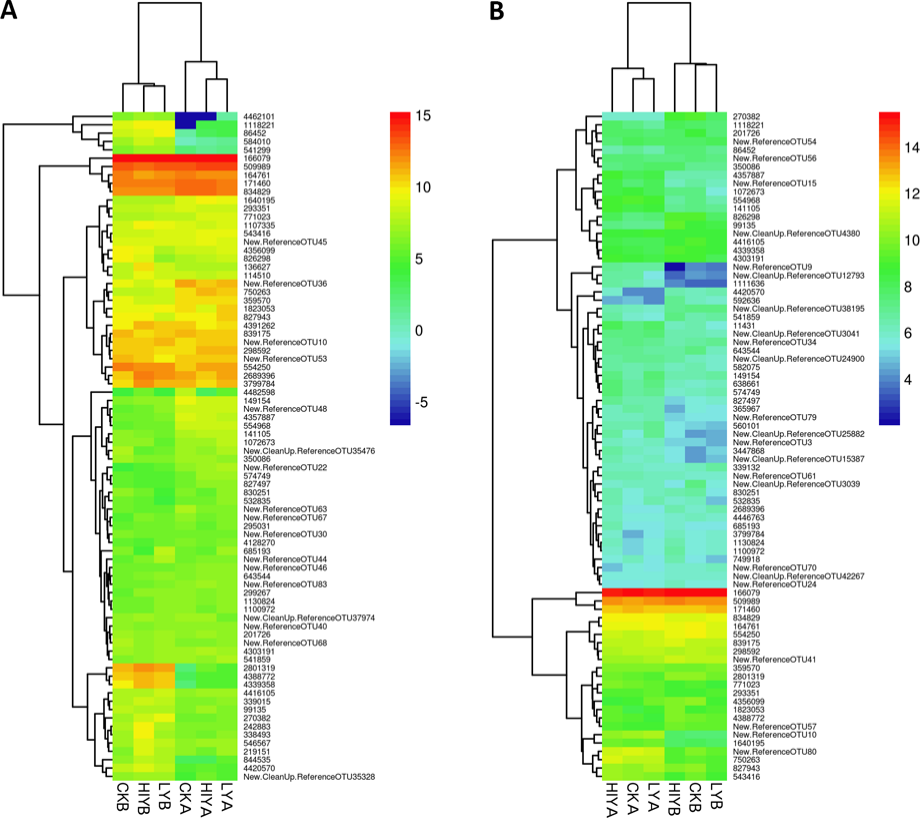
Heatmap showing the relative abundance of the top 80 OTUs of the microbiota. The figure describes autochthonous microbiota (A) and autochthonous microbiota (B) of Nile tilapia after 8 weeks of feeding with different diets. The microbial profiles of the 6 groups were clustered by complete linkage method.

For the autochthonous microbiota, the observed OTUs ranged from 1428 to 1678, and good’s coverage for all the samples was higher than 0.99. Neither yeast supplementation nor basal diet exerted a significant influence on the diversity of the autochthonous microbiota, as reflected by observed OTUs, Shannon index, and PD (S2 Table). Cluster analysis showed that the basal diet A and B formed two well-separated clusters, while live yeast and inactivated yeast exerted comparable effects on the microbiota with each basal diet (Fig 2). *Fusobacteria*, *Proteobacteria* and *Bacteroidetes* were the dominant groups in the microbiota. Yeast supplementation and basal diets had no significant effect on the abundance of each detected phylum. At the genus level, the abundance of *Deefgea* spp. was reduced in the live and inactive yeast groups, as compared to the control (*P* < 0.05) (Table 5).

**Table 5:** Genuses significantly changed in abundance by yeast supplementation.

	CK	LY	HIY	*P* value
*Deefgea* ^†^	0.97^a^	0.78^b^	0.78^b^	0.02
*Dechloromonas* ^‡^	0.80^a^	0.61^b^	0.90^a^	0.02
*Lactococcus* ^‡^	0.52^a^	0.64^b^	1.03^c^	0.003

Values are the mean relative abundance of a genus under two basal diets. Means sharing a common superscript letter (a, b, c) were not significantly different (P > 0.05).

^†^Genus from autochthonous microbiota.

^‡^Genus from allochthonous microbiota. CK, control check; LY, live baker’s yeast; HIY, heat-inactivated yeast.

Similar results were obtained for the allochthonous microbiota, with the observed OTUs ranging from 1295 to 1582 and good’s coverage higher than 0.99 for all the samples. The observed species richness was significantly lower in fish fed diet B compared to fish fed diet A (*P* < 0.05). Accordingly, a significantly lower PD was observed in the diet B group (*P* < 0.05), with the Shannon index marginally reduced (*P* = 0.097) (S3 Table). However, yeast supplementation had no significant influence on the diversity of allochthonous microbiota. Basal diet A and B again formed two separated clusters for the allochthonous microbiota. Under each basal diet, the alteration of microbiota induced by inactivated yeast was more pronounced than the one by live yeast (Fig 2). Similar to the autochthonous microbiota, *Fusobactetia*, *Proteobacteria* and *Bacteroidetes* were the dominant groups. Yeast supplementation did not have a significant effect on the abundance of the detected phyla. At the genus level, the abundance of *Dechloromonas* spp. and *Lactococcus* spp. was significantly influenced by yeast supplementation. *Lactococcus* spp. was enriched in both of the yeast supplementation groups compared to the control (*P* < 0.05), and the relative abundance in the inactivated yeast group was further up-regulated compared to the live yeast group (*P* < 0.05) (Table 5).

### Protection against *A*. *hydrophila* NJ-1 infection

Both live and inactive yeast significantly reduced alkaline phosphatase activity in the gut of tilapia after challenge by *A*. *hydrophila* NJ-1 (*P* < 0.001) (S3 Fig). Alkaline phosphatase activity was numerically lower in the live yeast group compared to the inactivated yeast group. Basal diet had no influence on the gut alkaline phosphatase activity, and no significant interaction between yeast supplementation and basal diet was observed (*P* > 0.10).

## Discussion

In this study, the contribution of secretory metabolites to the beneficial effects of baker’s yeast was investigated through comparison of fish fed diets supplemented with equal doses of live or heat-inactivated yeast, the latter being less susceptible to produce/secrete metabolites, as previously described [17]. It has been reported that some *Saccharomyces cerevisiae* strains may colonize the intestine of rainbow trout [29]. We conducted a preliminary experiment to investigate the fate of ingested live yeast in the intestine of tilapia by spreading plate method. Results showed that the yeast strain we used was only transient and the population of live yeast did not increase in the intestine of Nile tilapia (data not shown). Thus, we may rule out the proliferation of yeast in the intestine of fish, and the resulting higher concentration as a potential factor of differentiation between live and inactivated yeast. The difference between live and inactivated yeast therefore could be attributed to secretory metabolites.

Dietary supplementation of live baker’s yeast improved growth and feed utilization of Nile tilapia. Similar results have been reported previously in various fish species fed live yeast [5, 7–10], with the benefits attributed to improved diet and protein digestibility [5]. The growth promotion effect of yeast structural components like MOS, β-glucan and nucleic acids has been demonstrated in various fish species [12, 30, 31]. In contrast, the contribution of the secretory metabolites of yeast to the growth promotion effect has rarely been studied. Gao et al. suggested that phytase and oligosaccharides, as secretory metabolites in the yeast products, may contribute to improved Ca and P digestibility in broiler chicks [13]. In the present study, inactivated yeast did not significantly improve the growth and feed utilization of fish, indicating advantages of live yeast and the contribution of secretory metabolites to the growth of fish. However, the increased growth observed in the live yeast group relative to the inactivated one was marginal, suggesting that the secretory metabolites did not play a major role in the growth promotion effect. Although there was no direct comparison between live and inactive yeast, significantly improved growth performance with diets supplemented with inactivated yeast has previously been reported in fish [20, 22], supporting the major role of yeast structural components in the growth promotion effect. The lack of a significant growth improvement in fish fed inactivated yeast in our study might be due to the lower dose (0.1%) used here compared to previous studies (1%-2%) [20, 22].

Electron microscopy demonstrated that live yeast significantly improved microvilli length and density in the intestine of tilapia. As a component in the yeast cell wall, Mannan Oligosaccharides (MOS) were reported to improve villi and microvilli morphology in broiler chickens and pigs [32–35], as well as in various fish species [11, 36, 37]. MOS may prevent harmful bacteria from attaching to mannose residues on intestinal epithelial cells, therefore alleviating atrophy of (micro) villi due to inflammation from pathogens and their toxins. However, the present study showed that the inactive yeast, which contains equal amount of MOS to the live yeast, failed to exert beneficial effect on the microvilli morphology, suggesting that the yeast secretory metabolites acted as a main contributory factor to the improved microvilli morphology.

Live yeast supplementation significantly reduced the expression of *hsp70* in both intestine and head kidney of tilapia. HSP70 brings appropriate protection of protein structures, strengthens the immune system and stops apoptotic mechanisms [38], and its expression has been found to be related to stressful conditions in aquaculture, such as temperature oscillations, crowding, insufficient water quality, and improper diet [39]. Generally, *hsp70* expression decreases in fish given probiotics and coincides with improved welfare and growth [40–43]. In contrast to the live yeast feeding group, only a marginal reduction in *hsp70* expression was observed in the inactivated yeast group, indicating the importance of viability of yeast in the relief of stress status in fish.

Live yeast supplementation significantly reduced the expression of *tnfα* and tended to lower *il1β* expression in the intestine of tilapia, pointing to a down-regulation of inflammation in the intestine. Intestinal expression of *tgfβ* was significantly down-regulated by live yeast in fish fed diet A. TGFβ is known as an anti-inflammatory cytokines and down-regulated *tgfβ* expression is normally accompanied with up-regulation in proinflammatory cytokines [44]. However, simultaneous down-regulation in the expression of pro- and anti-inflammatory cytokines has also been reported, reflecting a generally down-regulated inflammation response [45]. Therefore, live yeast reduced the inflammation status in the intestine of tilapia. In contrast, the effect of inactivated yeast on expression of cytokines was minimal, suggesting that the secretory metabolites produced by the live yeasts contributed to the anti-inflammatory function. In accordance with our result, live yeast inhibited the expression of pro-inflammatory cytokines by enterotoxigenic *E*. *coli* treated porcine intestinal epithelial cells *in vitro*, with the effect attributed to secretory factors in the yeast [17]. The differential effect of live yeast on the expression of *tgfβ* in fish fed the two basal diets might be due to the difference in the metabolites produced by live yeasts, and needs further investigation. In head-kidney, the expression of *tnfα* and *il-1β* showed a down-regulated tendency in the yeast supplementation groups compared to the control, and the expression of *tgfβ* was significantly decreased in both live and inactivated yeast groups. Generally down-regulated expression of inflammation-related cytokines in head-kidney was also observed in common carp fed with *Saccharomyces cerevisiae* derived *β-*glucans [45]. Nevertheless, a stronger inflammation response was registered in the head-kidney of *β-*glucans fed common carp following the challenge by *A*. *salmonicida*, indicating an enhanced immunity [45]. Increased expressions of pro-inflammatory cytokines in the head-kidney of fish have been correlated with enhanced innate immunity upon probiotics feeding [46, 47, 48]. However, results in the present study suggest that other mechanisms beyond these cytokines may be involved in the immunity enhancement capacity of probiotics, as proposed by Panigrahi et al. [47].

Recent studies suggest that when the physiology of fish is altered through functional feeds the host becomes energy efficient rather than immunologically responsive, and a stronger immune response is achieved upon subsequent pathogen encounter [45, 49–50]. This rationale agrees with the observed expression of inflammation-related cytokines in response to live yeast in our study, where the host was not under the threat of pathogen attack. Also, reduced inflammation may direct more energy towards growth [51] and contribute to an enhanced transport of nutrients and metabolic processes in the intestine [45], leading to improved growth performance.

Beneficial effects of yeast products in ruminants are due to increased concentration of total and cellulolytic ruminal bacteria [52, 53], which may increase the availability of ME from diets, thereby increasing production. However, effects of yeast products on the intestinal microbiota of monogastric animals have been variable [13, 54]. Based on the results of 16S rRNA gene pysosequencing, supplementation of baker’s yeast, either alive or inactivated, exerted a subtle influence on autochthonous and allochthonous intestinal microbiota of tilapia fed with both diet A and B. The basal diets formed well-separated clusters for both autochthonous and allochthonous microbiota. The relative abundance of most genera detected was not significantly different between the yeast groups and control for both the autochthonous and allochthonous microbiota. Only *Deefgea* spp., *Dechloromonas* spp. and *Lactococcus* spp. showed significant difference in relative abundance due to yeast treatment. *Deefgea* spp. is a new genus including two species, i.e., *Deefgea rivuli* and *Deefgea chitinilytica*, both of which were isolated from aquatic environments, with *Deefgea chtinilytica* a chitinolytic bacteria that has been reported to be regularly isolated from freshwater ornamental fish [55]. *Dechloromonas* spp. is a genus from the phylum *Proteobacteria*, with some members having the ability to reduce perchlorate or degrade benzene. The potential function of these two genera in the intestinal microbiota of fish, as well as the implication of their abundance variation for the probiotic effect of baker’s yeast requires further investigation. The relative abundance of allochthonous *Lactococcus* spp. was significantly elevated in the yeast groups compared to the control, which is interesting because lactic acid bacteria including *Lactococcus* spp. are considered as beneficial bacteria [56, 57]. Increased lactic acid bacteria after yeast products/subcomponents supplementation has also been reported in other fish species [11, 20]. The extent to which this elevated abundance of *Lactococcus* spp. contributes to the observed beneficial effect of yeast deserves further assessment.

Gut alkaline phosphatase activity has been shown to be positively correlated with the number of *A*. *hydrophila* cells in the intestine after challenge and may reflect the resistance level of host against the pathogen (Liu et al. unpublished data). Protection of the host against pathogen was reflected by the significantly lower gut alkaline phosphatase activity in the baker’s yeast supplement groups. Similarly, increased diseases resistance after yeast supplementation has been reported in various fish species [7, 8, 10, 21]. The mechanism has been mainly attributed to the immunostimulating structural components, i.e., β-glucans, mannan oligosaccharides, chitin, as well as nucleotides [58]. In our study, the protection effect was marginally higher in the live yeast group compared with the inactivated yeast group, implying a minor involvement of the yeast secretory metabolites in the protection.

The formulations of the two basal diets were both extensively used in the aquaculture practice. In both diets, the inclusion of fishmeal was relatively low, which was replaced by soybean meal and rapeseed meal. Although the level of soybean meal was higher in diet B, no intestinal inflammation was observed, which may be attributed to the low inclusion of soybean meal in this diet. Moreover, diet B generally gave improved growth, feed utilization, microvilli morphology, as well as intestinal stress status (lower *hsp70* expression), indicating a more balanced nutrition of diet B compared with diet A. Nevertheless, there were no difference in disease resistance between fish groups fed diet A and diet B, implying that the advantage of diet B mainly involves nutrition, with no effect on the host immunity. Apart from the *tgfβ* expression in intestine, there were no interactions between yeast treatment and basal diets for all the parameters evaluated, indicating that the beneficial effects of yeast, as well as the advantage of live yeast, don’t depend on the protein profiles of the basal diets and the different intestinal microbiota they induced.

In conclusion, the present study showed the advantages of live yeast as a dietary supplement for Nile tilapia, as indicated by the improved gut microvilli morphology, reduced *hsp70* expression level, as well as the generally down-regulated intestinal inflammation status, which suggests a role for yeast secretory metabolites. However, the observed improvement at the microscopic/molecular level did not translate into evident improvement in growth performance and disease resistance, and only a marginal increase was observed. Therefore, the secretory metabolites might not play a major role in growth promotion and disease protection effects of yeast, as compared to the structural components, which might be due to the limited dose provided by the live yeast in the host or the duration of feeding. Nevertheless, the beneficial effects and modes of action of yeast secretory metabolites deserve further study, which may lead to the development of novel functional dietary supplements for the replacement of antibiotics.

## Supporting Information

S1 Fig The original SEM images of the gut microvilli of tilapia fed different diets. (DOCX)

S2 Fig Intestinal expression of *tgfβ* under different basal diets. The expression of *tgfβ* in the intestine was separately analyzed under different basal diets with one way ANOVA. Under each diet, means sharing a same superscript letter were not significantly different.
(DOCX)

S3 Fig The gut alkaline phosphatase activity of fish after challenge with *Aeromonas hydrophila* NJ-1 (n = 8). The superscript letters describe the main effect of yeast supplementation from two way ANOVA and Tukey post hoc, with groups sharing the same letter not significantly different (*P* > 0.05).
(DOCX)

S1 Table Sequences of oligonucleotide primers for *q*PCR. (DOC)

S2 Table Effect of yeast and basal diets on the diversity of autochthonous microbiota of Nile tilapia. (DOCX)

S3 Table Effect of yeast and basal diets on the diversity of allochthonous microbiota of Nile tilapia. (DOCX)
