# Correction: A Comparison of the Beneficial Effects of Live and Heat-Inactivated Baker's Yeast on Nile Tilapia: Suggestions on the Role and Function of the Secretory Metabolites Released from the Yeast

**DOI:** 10.1371/journal.pone.0151207

**Published:** 2016-03-10

**Authors:** Chao Ran, Lu Huang, Zhi Liu, Li Xu, Yalin Yang, Philippe Tacon, Eric Auclair, Zhigang Zhou

There is an error in the caption for Fig 2. Please see the complete, correct Fig 2 here.

**Fig 2:**
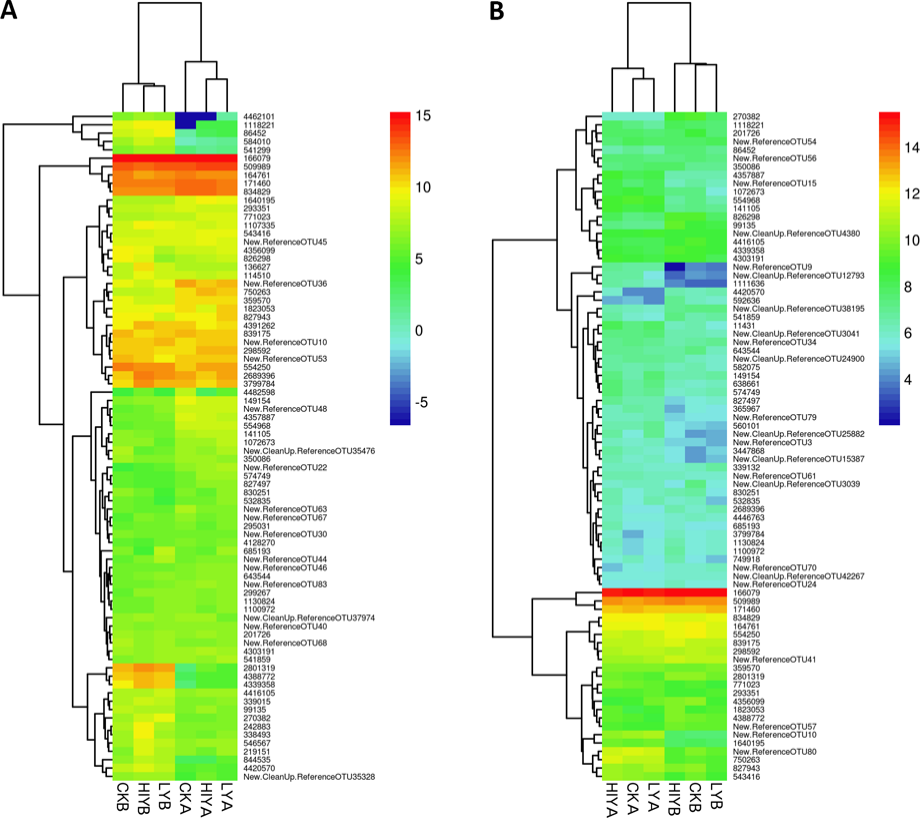
Heatmap showing the relative abundance of the top 80 OTUs of the microbiota. The figure describes autochthonous microbiota (A) and allochthonous microbiota (B) of Nile tilapia after 8 weeks of feeding with different diets. The microbial profiles of the 6 groups were clustered by complete linkage method.

## References

[1] Ran C, Huang L, Liu Z, Xu L, Yang Y, Tacon P, et al (2015) A Comparison of the Beneficial Effects of Live and Heat-Inactivated Baker’s Yeast on Nile Tilapia: Suggestions on the Role and Function of the Secretory Metabolites Released from the Yeast. PLoS ONE 10(12): e0145448 doi:10.1371/journal.pone.0145448 26696403 10.1371/journal.pone.0145448 PMC4690590

