# Supplementary material for: A Comparison of the Beneficial Effects of Live and Heat-Inactivated Baker’s Yeast on Nile Tilapia: Suggestions on the Role and Function of the Secretory Metabolites Released from the Yeast
Source: PLoS One. 2015 Dec 22;10(12):e0145448. doi: 10.1371/journal.pone.0145448 (PMC4690590; doi:10.1371/journal.pone.0145448)
Supplement: S1 Fig — (DOCX) [file pone.0145448.s001.docx]

**S1 Fig. The original SEM images of the gut microvilli of tilapia fed different diets.**
