# Supplementary material for: A Comparison of the Beneficial Effects of Live and Heat-Inactivated Baker’s Yeast on Nile Tilapia: Suggestions on the Role and Function of the Secretory Metabolites Released from the Yeast
Source: PLoS One. 2015 Dec 22;10(12):e0145448. doi: 10.1371/journal.pone.0145448 (PMC4690590; doi:10.1371/journal.pone.0145448)
Supplement: S2 Fig — The expression of tgfβ in the intestine was separately analyzed under different basal diets with one way ANOVA. Under each diet, means sharing a same superscript letter were not significantly different. (DOCX) [file pone.0145448.s002.docx]

**
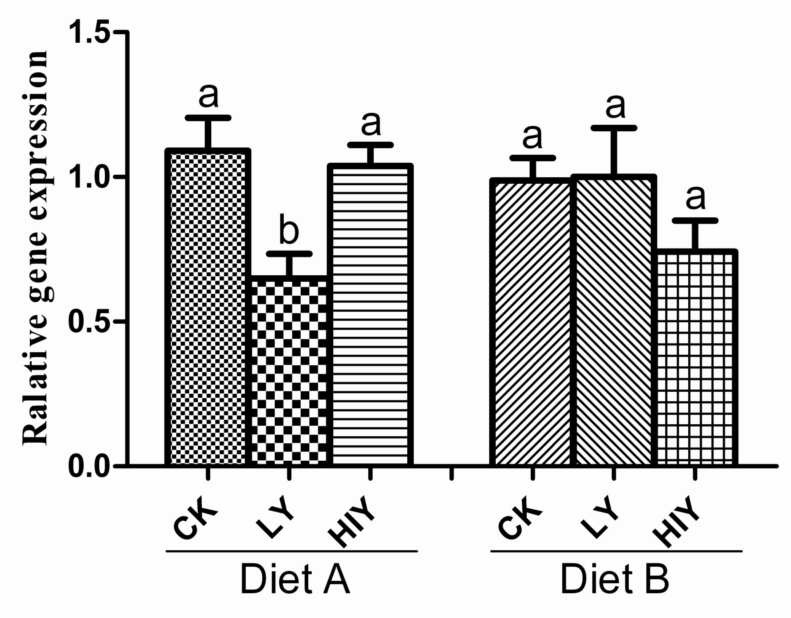
**

**S2 Fig. Intestinal expression of *tgfβ* under different basal diets.** The expression of *tgfβ* in the intestine was separately analyzed under different basal diets with one way ANOVA. Under each diet, means sharing a same superscript letter were not significantly different.
