# Supplementary material for: A Comparison of the Beneficial Effects of Live and Heat-Inactivated Baker’s Yeast on Nile Tilapia: Suggestions on the Role and Function of the Secretory Metabolites Released from the Yeast
Source: PLoS One. 2015 Dec 22;10(12):e0145448. doi: 10.1371/journal.pone.0145448 (PMC4690590; doi:10.1371/journal.pone.0145448)
Supplement: S3 Fig — The superscript letters describe the main effect of yeast supplementation from two way ANOVA and Tukey post hoc, with groups sharing the same letter not significantly different (P > 0.05). (DOCX) [file pone.0145448.s003.docx]

**
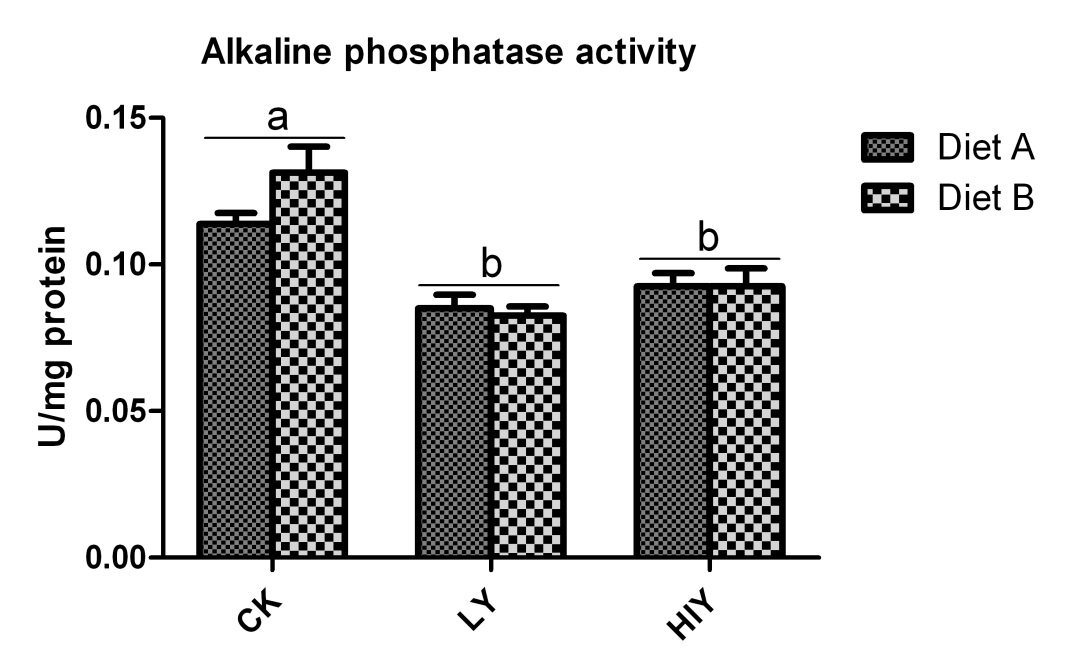
**

**S3 Fig. The gut alkaline phosphatase activity of fish after challenge with *Aeromonas hydrophila* NJ-1 (n=8).** The superscript letters describe the main effect of yeast supplementation from two way ANOVA and Tukey post hoc, with groups sharing the same letter not significantly different (*P* > 0.05).
