# Supplementary material for: A Comparison of the Beneficial Effects of Live and Heat-Inactivated Baker’s Yeast on Nile Tilapia: Suggestions on the Role and Function of the Secretory Metabolites Released from the Yeast
Source: PLoS One. 2015 Dec 22;10(12):e0145448. doi: 10.1371/journal.pone.0145448 (PMC4690590; doi:10.1371/journal.pone.0145448)
Supplement: S1 Table — (DOC) [file pone.0145448.s004.doc]

**S1 Table. Sequences of oligonucleotide primers for *q*PCR**

| Target genes | Primers | Sequence (5’-3’) | Reference^*^ |
| --- | --- | --- | --- |
| Act-β | F | GCTACTCCTTCACCACCACAG | JF957365 |
|  | R | CGTCAGGCAGCTCGTAACTC |  |
| HSP70 | F | TGCCTTTGTCCAGACCGTAG | JF957370 |
|  | R | GTGTCCAACGCTGTCATCAC |  |
| IL-1β | F | TGCACTGTCACTGACAGCCAA | JF957374 |
|  | R | ATGTTCAGGTGCACTTTGCGG |  |
| TNF-α | F | CTTCCCATAGACTCTGAGTAGCG | JF957367 |
|  | R | GAGGCCAACAAAATCATCATCCC |  |
| TGF-β  V3-V4 of  16s rRNA | F | TGCGGCACCCAATCACACAAC | JF957373  (1)  (2) |
|  | R | GTTAGCATAGTAACCCGTTGGC |  |
|  | 341F | CCTACGGGAGGCAGCAG |  |
|  | 806R | GGACTACCVGGGTATCTAAT |  |

**^*^** NCBI accession No.

**References**

1. Muyzer G, De Waal E C, Uitterlinden A G. Profiling of complex microbial populations by denaturing gradient gel electrophoresis analysis of polymerase chain reaction-amplified genes coding for 16S rRNA. Appl Environ Microb 1993;59(3):695-700.
2. Srinivasan S, Hoffman N G, Morgan M T, et al. Bacterial communities in women with bacterial vaginosis: high resolution phylogenetic analyses reveal relationshipof microbiota to clinical criteria. PloS ONE 2012;7(6):e37818.
