# Supplementary material for: A Comparison of the Beneficial Effects of Live and Heat-Inactivated Baker’s Yeast on Nile Tilapia: Suggestions on the Role and Function of the Secretory Metabolites Released from the Yeast
Source: PLoS One. 2015 Dec 22;10(12):e0145448. doi: 10.1371/journal.pone.0145448 (PMC4690590; doi:10.1371/journal.pone.0145448)
Supplement: S2 Table — (DOCX) [file pone.0145448.s005.docx]

**S2 Table. Effect of yeast and basal diets on the diversity of autochthonous microbiota of Nile tilapia**

|  | Yeast | | |  | Basal diet | |  | *P* value | |
| --- | --- | --- | --- | --- | --- | --- | --- | --- | --- |
|  | CK | LY | HIY |  | A | B |  | Yeast | Basal diet |
| OTU | 1491.0 | 1655.0 | 1523.5 |  | 1496.7 | 1616.3 |  | 0.14 | 0.10 |
| Shannon | 4.4 | 4.7 | 4.8 |  | 4.5 | 4.8 |  | 0.52 | 0.30 |
| PD | 60.2 | 66.9 | 61.3 |  | 61.8 | 63.7 |  | 0.15 | 0.37 |

Values are means of two or three groups for ‘Yeast’ and ‘Basal diet’ unit cells, respectively. Analysis was conducted by two way ANOVA without replication.
