# Supplementary material for: A Comparison of the Beneficial Effects of Live and Heat-Inactivated Baker’s Yeast on Nile Tilapia: Suggestions on the Role and Function of the Secretory Metabolites Released from the Yeast
Source: PLoS One. 2015 Dec 22;10(12):e0145448. doi: 10.1371/journal.pone.0145448 (PMC4690590; doi:10.1371/journal.pone.0145448)
Supplement: S3 Table — (DOCX) [file pone.0145448.s006.docx]

**S3 Table. Effect of yeast and basal diets on the diversity of allochthonous microbiota of Nile tilapia**

|  | Yeast | | |  | Basal diet | |  | *P* value | |
| --- | --- | --- | --- | --- | --- | --- | --- | --- | --- |
|  | CK | LY | HIY |  | A | B |  | Yeast | Basal diet |
| OTU | 1410.5 | 1399.0 | 1487.5 |  | 1515.7 | 1349.0 |  | 0.25 | 0.04 |
| Shannon | 4.3 | 4.2 | 4.5 |  | 4.6 | 4.1 |  | 0.42 | 0.097 |
| PD | 60.9 | 62.0 | 64.2 |  | 65.9 | 58.9 |  | 0.40 | 0.047 |

Values are means of two or three groups for ‘Yeast’ and ‘Basal diet’ unit cells, respectively. Analysis was conducted by two way ANOVA without replication.
